# Feasibility of using nonflat photon beams for whole‐breast irradiation with breath hold

**DOI:** 10.1120/jacmp.v15i1.4397

**Published:** 2014-01-06

**Authors:** Yuenan Wang, Andrew Vassil, Rahul Tendulkar, John Bayouth, Ping Xia

**Affiliations:** ^1^ Department of Radiation Oncology Cleveland Clinic Cleveland OH; ^2^ Department of Radiation Oncology Iowa University Iowa City IA 52242 USA

**Keywords:** flattening filter‐free, nonflat photon beams, forward‐planning, direct aperture optimization, breast radiotherapy

## Abstract

Removing a flattening filter or replacing it with a thinner filter alters the characteristics of a photon beam, creating a forward peaked intensity profile to make the photon beam nonflat. This study is to investigate the feasibility of applying nonflat photon beams to the whole‐breast irradiation with breath holds for a potential of delivery time reduction during the gated treatment. Photon beams of 6 MV with flat and nonflat intensity profiles were commissioned. Fifteen patients with early‐stage breast cancer, who received whole‐breast radiation without breathing control, were retrospectively selected for this study. For each patient, three plans were created using a commercial treatment planning system: (a) the clinically approved plan using forward planning method (FP); (b) a hybrid intensity‐modulated radiation therapy (IMRT) plan where the flat beam open fields were combined with the nonflat beam IMRT fields using direct aperture optimization method (mixed DAO); (c) a hybrid IMRT plan where both open and IMRT fields were from nonflat beams using direct aperture optimization (nonflat DAO). All plans were prescribed for ≥95% of the breast volume receiving the prescription dose of 50 Gy (2.0 Gy per fraction). In comparison, all plans achieved a similar dosimetric coverage to the targeted volume. The average homogeneity index of the FP, mixed DAO, and nonflat DAO plans were 0.882±0.024, 0.879±0.023, and 0.867±0.027, respectively. The average percentage volume of $V_{105}$ was 57.66%±5.21%, 34.67%±4.91%, 41.64%±5.32% for the FP, mixed, and nonflat DAO plans, respectively. There was no significant difference (p>0.05) observed for the defined endpoint doses in organs at risk (OARs). In conclusion, both mixed DAO and nonflat DAO plans can achieve similar plan quality as the clinically approved FP plan, measured by plan homogeneity and endpoint doses to the ORAs. Nonflat beam plans may reduce treatment time in breath‐hold treatment, especially for hypofractionated treatment.

PACS number: 87.55

## I. INTRODUCTION

Breath‐hold treatment techniques for patients with breast cancer can significantly reduce radiation dose to the heart, especially for patients with left side breast cancer, decreasing the probability of late cardiovascular toxicities.^1^, ^2^, ^3^ Using conventional dose rates, typically from 200 MU/min to 600 MU/min, the breath‐hold treatment can protract the delivery time, increase patient discomfort, and potentiate delivery errors due to inherent irregularities in breath holds. By removing the flattening filter or replacing it with a thin filter, the nonflat radiation beam creates a forward peaked intensity profile with a dose rate at the central axis approximately three to five times higher than that of the conventional flat beam. This drastically increased dose rate may substantially shorten the beam‐on time, especially for patients receiving treatments under breathing holds, or automatic gating.

For small fields, the beam profiles of nonflat beams are similar to those of conventional flat beams, but major differences are observed in the beam profiles of fields that are greater than 4‐6 cm. For whole‐breast radiation, one of the most frequent treatments in radiotherapy, field sizes greater than 4‐6 cm are often used. Due to the characteristics of the nonflat beams, it may be challenging to achieve uniform dose distribution for whole‐breast irradiation. The use of nonflat beams may pose a challenge for the commonly utilized field‐in‐field (or forward‐planning) technique.^4^, ^5^, ^6^ Inverse plan optimization may be necessary when nonflat beams are used for treatment. There are conflicting reports regarding inverse planning for breast treatment using flat beams.^7^, ^8^, ^9^ Mayo et al.^(9)^ reported that IMRT plans created with an inverse planning method for whole‐breast radiation result in worse dose homogeneity inside the breast, higher maximal dose outside the target, and 2.3 times more monitor units (MUs) than in the forward‐planned intensity‐modulated radiation therapy (FP‐IMRT) plans. Ahunbay et al.^(7)^ reported that direct aperture optimization (DAO) IMRT, where the delivery parameters (i.e., number of segments, shapes, and weights) are directly considered during the optimization process, can achieve the similar plan quality as FP‐IMRT plans. Using a different treatment planning system, Ahunbay and colleagues and Descovich et al.^(8)^ reported that a mixed open field with intensity‐modulated fields, which they called hybrid direct aperture‐based IMRT plans, achieved similar the plan quality as FP‐IMRT techniques. The purpose of this study is to investigate whether a comparable plan quality can be achieved with the nonflat beams using the inverse planned IMRT technique when compared to the plans with the conventional flat beams using the forward‐planning technique for breast treatment with breath holding.

## II. MATERIALS AND METHODS

### A. Patient selection

Fifteen patients with early‐stage breast cancer were retrospectively and randomly selected for this feasibility study, which was approved by our local institution review board. Among the 15 patients, seven had left‐breast cancer and eight had right‐breast cancer. Based on the breast volume, five patients were categorized to the small‐size breast group (781±85 cc), five to the medium‐size breast group (1149±154 cc), and five to the large‐size breast group (1751±150 cc). The average breast size of three groups was significantly different (all p<0.01).

### B. Treatment plans

The simulation process for all cases began with the setup of the patients on a CT simulator (AcQSim; Philips Healthcare, Cleveland, OH), where the superior, inferior, medial, and lateral borders were determined by radiation oncologists based on the breast tissue volume, setup margin, and amount of lung volumes exposed to radiation. The 3 mm thick CT slices were acquired and transferred to the Pinnacle treatment planning system (Pinnacle 8.0m, Philips Healthcare System).

The 6 MV and 10 MV flat photon beams (Artiste, Siemens Medical Systems, Erlangen, Germany) were commissioned in the Pinnacle treatment planning system (TPS) for clinical use in our institution. The 6 MV nonflat photon beams (ONCOR, Siemens Medical Systems) were commissioned for clinical use in the Pinnacle TPS from the University of Iowa. Both flat and nonflat beams were calibrated using a fixed SSD at 100 cm for $10\times 10\;{\text{cm}}^{2}$ field size at the depth of 1.5 cm for 1 cGy/MU.

For each patient, three plans were created. The first was the original clinically approved plan using forward‐planning (FP) with mixed 6 MV and 10 MV flat beams. The intact breast was treated with a pair of tangential flat photon fields to encompass the entire breast, sparing as much lung and heart as possible. Additional wedges or multiple segments defined by the multileaf collimator (MLC) were manually created for the medial and lateral fields to achieve dose homogeneity while maintaining dose coverage to the targeted volume.^(10)^ The second plan consisted of two open tangential fields with flat 6 MV beams combined with two tangential IMRT fields with nonflat 6 MV beams, where the IMRT fields were optimized with the direct machine parameter optimization (DMPO). The third plan was similar to the second plan by replacing the two open tangential fields with the nonflat 6 MV beam and using DAO IMRT planning. The detailed DAO planning procedure was described by Descovich et al.^(8)^ and referred to as hybrid direct aperture‐based optimization (DAO). In this study, we referred to plans created by the second planning method as the mixed DAO plans, and to plans created by the third planning method as the nonflat DAO plans. All plans were created using the same isocenter, located about 2 cm from the chest wall inside the breast tissue.

**Figure 1:**
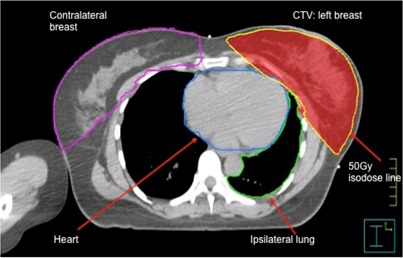
Illustration of the contours of the CTV and other OARs.

For the original clinically approved FP plan, the clinical target volume (CTV) was not specifically delineated for planning. For the purpose of this study, the prescription isodose line (50 Gy isodose line) was converted as the clinical target volume (CTV), excluding 5 mm external skin surface.^(8)^ The planning target volume is the same as the CTV. The organs at risk (OARs), such as the ipsilateral lung, contralateral breast, and heart for left side breast cancer, were also contoured for plan evaluation. Figure 1 illustrates details of the CTV contour, contours of the OARs, and contralateral breast for a selected patient with left side breast cancer. All three types of plans were created using Pinnacle version 8.0.

### C. Plan evaluation

All plans were prescribed for ≥95% of the PTV receiving total 50 Gy in 25 fractions. Homogeneity index (HI) was used to evaluate plan quality, which was the prescription dose divided by the maximum dose, defined as:

(1) $$\text{HI}=D_{\text{Rx}}/D_{\text{max}}$$

where $D_{Rx}$ is the prescription dose, which is 50 Gy for all plans, and $D_{\text{max}}$ is the maximum point dose of each plan.

Conformality index (COIN)^(11)^ was defined as:

(2) $$\text{COIN}=({\text{PTV}}_{\text{ref}}/\text{PTV})\times ({\text{PTV}}_{\text{ref}}/{\text{Tissue}}_{\text{ref}})$$

where ${\text{PTV}}_{\text{ref}}$ was the volume of the portion of PTV enclosed by the prescription isodose line, and ${\text{Tissue}}_{\text{ref}}$ was the total tissue volume enclosed by the prescription isodose line Thus, the higher HI, the more uniform the plan; the closer COIN to unity, the more conformal the plan.

$V_{105}$ was the percentage of the tissue volume receiving 105% of the prescription dose, which could be used to measure the volume of the hot spot. Clinically, it was desired to keep HI close to 90% and minimize $V_{105}$. The maximum dose to 1 cc (i.e., $D_{1\text{cc}}$) was also calculated, which represented a volume specific hot spot, instead of the maximum point dose. In addition, dose to OARs were evaluated for selected critical structures, such as $V_{20\text{Gy}}$ to ipsilateral lung (the percent volume receiving 20 Gy), $V_{1\text{Gy}}$ to the contralateral breast, and $V_{25\text{Gy}}$ to heart for left breast. The plans were also evaluated for their delivery efficiency based on the total MUs. All parameters from three types of plans were compared using Wilcoxon nonparametric signed‐rank test with p‐value <0.05 being considered as significantly different.

## III. RESULTS

### A. Dosimetry characteristics

**Figure 2:**
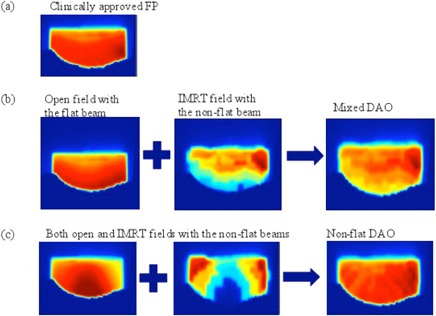
Illustration of fluence maps for a medial field from (a) clinically approved FP, (b) mixed DAO, and (c) nonflat DAO plans.

**Figure 3:**
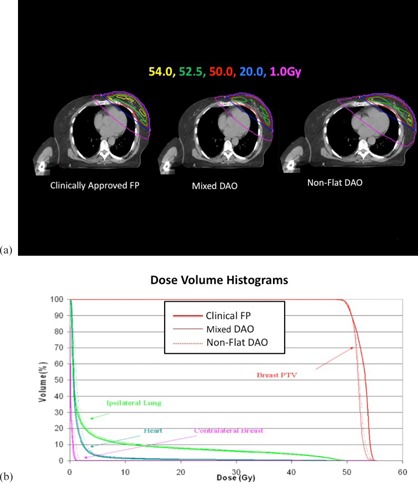
Axial images (a) with various isodose lines for a selected patient with left‐side breast cancer; (b) dose‐volume histograms for the clinical FP, mixed DAO, and nonflat DAO plans.

Figure 2 shows a typical fluence map of a medial field from the FP, mixed DAO, and nonflat DAO plans for a representative patient. In Fig. 2, a relatively uniform fluence was achieved by three planning methods. Figures 3(a) and (b) show the resultant isodose lines and dose‐volume histograms (DVHs) of the three plans. Similar dose distributions in the PTV and critical structures for the three types of plans were observed. As shown in Fig. 3(a), on this particular image of the selected patient, the 54 Gy isodose line was substantially smaller in both types of DAO plans, when compared to the FP plan. This observation was true for all 15 patients with small, median, and large treatment volumes.

For the FP, mixed DAO, and nonflat DAO plans, Table 1 lists the average homogeneity index, conformality index, hotspot endpoints ($D_{1\text{cc}}$ and $V_{105}$), total MUs, and number of segments. Statistically, there were significant differences in homogeneity index (HI) between clinically FP plans and nonflat DAO (p<0.001), and between mixed DAO and nonflat DAO (p<0.01). There was no significant difference in the conformality index among all plans (all p>0.05). There was no significant difference in $D_{1\text{cc}}$ between the FP and nonflat DAO plans (p>0.05), but $D_{1\text{cc}}$ was significantly lower in mixed DAO plans than that of nonflat DAO plans. The $V_{105}$ of clinically approved FP plans was significantly (p<0.002) higher than that of mixed nonflat DAO plans. The $V_{105}$ of mixed DAO plans was also significantly (p<0.001) lower than that of nonflat DAO plans. The average MU for nonflat DAO was 35% more MUs than that of the mixed DAO plans, which was 21% higher than the average MU for the FP plans. Table 2 compares selected endpoints for organs at risk (OARs), such as the ipsilateral lung, heart, and contralateral breast. There was no significant difference (p>0.05) in ipsilateral lung $V_{20\text{Gy}}$ heart $V_{25\text{Gy}}$, or contralateral breast $V_{1\text{Gy}}$.

**Table 1:** Comparison of the average (standard deviation) plan herterogneity index, conformal index, hotspot endpoints, MU, and number of segments

	*HI (%)*	*CI (%)*	$D_{1cc}\;(Gy)$	$V_{105}\;(\%)$	*MU*	*Segment*
Clinically approved FP	0.88 (0.019)	0.95 (0.008)	56.01 (0.35)	57.7 (5.2)	2343±40	7±3
Mixed DAO	0.88 (0.021)	0.95 (0.005)	55.86 (0.46)	34.7 (4.9)	249±26	11±1
Nonflat DAO	0.87 (0.024)	0.94 (0.004)	56.51 (0.5)	41.6 (5.3)	396±49	12±0

a FP=forward planning; DAO=direct aperture optimization; HI=heterogenity index; CI=conformal index; MU=monitor unit.

**Table 2:** The average (standard deviation) endpoint doses of the ipsilateral lung, heart, and contraleral breast for three types of plans

	*Ipsilateral Lung* $V_{20\text{Gy}}$	*Heart* $V_{25\text{Gy}}$ *(left breast)*	*Contralateral Breast* $V_{1\text{Gy}}$
Clinically approved FP	10.0±2.7% ^a^	1.9±2.4% ^a^	9.2±10.2% ^a^
Mixed DAO	10.4±2.9%	2.0±2.4%	8.9±7.6%
Non‐flat DAO	10.5±2.9%	2.0±2.4%	11.5±7.3%

a ^a^ P>0.05.

FP=forward planning; DAO=direct aperture optimization

## IV. DISCUSSION

The hybrid DAO technique has been proposed and proved to be feasible to generate uniform dose distributions in whole‐breast irradiation with conventional flat beams.^(8)^ Our study is focused on the feasibility of applying the DAO technique with nonflat photon beams to achieve dose uniformity and on the evaluation of the dosimetric performance of nonflat photon beams in whole‐breast irradiation.

The fluence map of the three plans illustrated in Fig. 2 demonstrated that the hybrid DAO IMRT method using nonflat beams is capable of obtaining dose uniformity in whole‐breast irradiation by combining the open nonflat fields with the IMRT fields. The plan homogeneity and conformity of mixed beam DAO plans were comparable to the clinical FP plans. The specific endpoints, such as $D_{1\text{cc}}$ and $V_{105}$, were improved in mixed beam DAO plans when compared to clinical FP plans. For conventional fractionation treatment without breath hold, the use of the nonflat beam may not be necessary if all energies of flat beams are available. In practice, one may be forced to make a choice of not having all photon energies in both flat and nonflat beams. In this case, the mixed beam DAO plans have dosimetric advantage over nonflat DAO plans to reduce potential imperfect matches among all subfields (or segements). For treatment with breath‐hold and hypofractionation treatments,^(12)^ number of breath holds could be a dominant fact to consider, and nonflat beam DAO plans delivered with a very high dose rate may save the treatment time by reducing the number of breath holds. In this study, we only used 6 MV flat and 6 MV nonflat beams for mixed and nonflat DAO plans. The 6 MV flat and 6 MV nonflat beams were the only photon beams commissioned for this study. With possible choices of higher energy nonflat beams, mixing a flat 6 MV beam with a nonflat higher energy beam might even further improve the dose uniformity for mixed and nonflat DAO plans.

With the same treatment planning system as we used for this study, it has been reported that MUs of the IMRT plans using the nonflat beams were much lower than that of the corresponding IMRT plans using the flat beams from Varian 23EX machine (Varian Medical Systems, Palo Alto, CA).^(13)^ It should be noted that the dose to MU conversion in reference was set to 1.99 cGy/MU for 6 MV nonflat beam and 3.62 cGy/MU for 18 MV nonflat beam. In our study, we set the dose to MU conversion to 1 cGy/MU for the flat and nonflat beams. Because of using more segments and smaller radiation aperture sizes in the mixed DAO and nonflat DAO plans, we observed 21% more MUs for the mixed DAO plans and 63% more MU for the nonflat DAO plans when compared to the clinical approved FP plans using the flat beams.

Despite the increase in MUs for the nonflat beam plans, the total delivery time, especially the number of breath holds for each treatment, may reduce because of a substantially high dose rate. The impact of the total delivery time depends on a specific delivery system. Beside the total MU and the dose rate, the IMRT delivery method (e.g., step‐and‐shoot versus slide window) is another confounding factor. Even for the step‐and‐shoot delivery method, the delivery time can vary from different models of linear accelerators from the same manufacturer, or linear accelerators from the different manufacturers. With a specific linear accelerator from Siemens Medical Solution (the linear accelerator used for this study), the MLC leaf positions in each segment (or subfield) are verified prior to radiation, and the jaw positions are conformal to each segment to reduce radiation leakage through MLCs, thus consuming a substantial portion of the “beam‐on” time. Strictly speaking, this time is not a part of “beam‐on” time because the beam is “on hold” during this time. For practical reasons, the transition time between segments is still considered as a part of the “beam‐on” time. For other vendors (e.g., Varian), the transition time between segments is much shorter because the MLC positions are not verified but recorded in a log file and because the jaws are parked at a stationary positions. When the transition time between segments becomes negligible, the total MU and the dose rate used for delivery are the dominant factors to calculate (or estimate) the beam‐on time. Hypothetically, if the clinical FP plans were delivered with 600 MU/min dose rate without taking into account the transition time between segments, 243 MU would require 24 seconds to deliver all segments in two beam angles. If nonflat DAO plans were delivered with 1600 MU/min dose rate, 396 MU would require 15 seconds to deliver all segments in two beam angles.

In addition to significant increase of the dose rate, nonflat beams possess other dosimetric advantages such as reducing head scatter, decreasing penumbra, and lowering the out‐of‐field dose, shown by Monte Carlo simulations and experimental measurements.^14^, ^15^ Comparing the peripheral dose in intensity‐modulated radiation (IMRT) plans for pediatric intracranial tumors delivered with nonflat beams versus with conventional flat beams, Cashmore^(15)^ found that IMRT with nonflat beams reduced leakage‐related doses by up to 70%. Kragl et al.^(16)^ also reported similar results. For high‐energy beams, Kry et al.^(17)^ reported that fewer neutrons are produced by the nonflat beams. In this study, we did not specifically explore these dosmimetric advantages. Further studies are needed to explore how these dosimetric advantages translate into the specific clinical benefits for the whole‐breast irradiation. *In vivo* measurements, such as skin dose, dose to the contralateral breast, and scattering dose‐to‐distance organs, are needed.

## IV. CONCLUSIONS

By comparing three different types of plans with the flat and nonflat beams in whole‐breast irradiation quantitatively, we concluded that both mixed DAO and nonflat DAO plans can achieve dosimetric equivalence as the clinically approved FP plan. For patients treated with breath hold and hypofractionation, using nonflat beams with high dose rates, the treatment time might be reduced. The reduction of the treatment time is only a prediction, and clinical confirmation is required.

## ACKNOWLEDGMENTS

This research is supported in part by a research grant from Siemens Medical Solution.
